# Isolated Population of Proboscis Monkeys and Their Status in Sulaman Lake Forest Reserve, Sabah, Malaysia

**DOI:** 10.21315/tlsr2025.36.1.5

**Published:** 2025-03-30

**Authors:** Mohd. Aminur Faiz Suis, Marrynah Matami, Miyabi Nakabayashi, Aini Hasanah Abd Mutalib, Fazidah Ismail, Fadzilah Awang Kanak, Joseph Tangah

**Affiliations:** 1 Research Management Centre, Universiti Malaysia Sabah, 88400 Kota Kinabalu, Sabah, Malaysia; 2 Forest Research Centre, Sabah Forestry Department, P.O. Box 1407, 90715 Sandakan, Sabah, Malaysia; 3 Graduate School of Integrated Sciences for Life, Hiroshima University, Kagamiyama, Higashi-Hiroshima City, Hiroshima 739-8528, Japan; 4 Institute of Tropical Biodiversity and Sustainable Development, Universiti Malaysia Terengganu, 21030 Kuala Nerus, Terengganu, Malaysia; 5 Preparatory Centre for Science and Technology, Universiti Malaysia Sabah, 88400 Kota Kinabalu, Sabah, Malaysia

**Keywords:** Malaysian Borneo, Mangrove Fragmentation, Colobine, Relict Population, Sulaman Bay, Malaysia Borneo, Fragmentasi Hutan Bakau, Kolobin, Populasi Relik, Teluk Sulaman

## Abstract

Proboscis monkeys are largely confined along the eastern coastal zone of Sabah, Malaysia. Sulaman Lake Forest Reserve (SLFR) is the only Class V Mangrove Forest Reserve in the forestry district of Kota Kinabalu, Sabah. This study is in accordance with the conservation strategies outlined in the Sabah Proboscis Monkey Action Plan (2019–2028), which is to focus on areas that have not been surveyed. Prior to this study, the status of proboscis monkeys in this reserve had not been scientifically documented. In addition, the habitat of this colobine species along the coastal zone of Sulaman Bay will be impacted by the construction of Pan Borneo Highway (Work Package 9). Hence, we intend to shed light on the status of proboscis monkeys in the SLFR including local threats to their population. This study discovered a small and isolated relict population of proboscis monkeys through a boat survey approach, which was also derived from interview sessions with local communities. We found that the degradation of mangrove forest integrity is a major threat to this population in the SLFR. It is hoped that this study will trigger more research, especially population size estimation, because such baseline information is crucial in the formulation of effective conservation programmes.

## Abstract (fr)

**Abstrak:**

Sebahagian besar populasi monyet bangkatan di Sabah, Malaysia wujud di sepanjang zon pantai timur. Hutan Simpan Tasik Sulaman (HSTS) merupakan satu-satunya Hutan Kelas V iaitu Hutan Simpan Bakau di daerah perhutanan Kota Kinabalu, Sabah. Kajian ini adalah selari dengan strategi pemuliharaan dalam Pelan Tindakan Monyet Bangkatan Sabah (2019–2028) iaitu memfokuskan kawasan yang belum pernah ditinjau. Sebelum kajian ini dijalankan, status monyet bangkatan di HSTS belum pernah didokumentasi secara saintifik. Habitat monyet ini di sepanjang kawasan pantai Teluk Sulaman juga akan terkesan dengan pembinaan Lebuh Raya Pan Borneo (Pakej Kerja 9). Justeru kami ingin menilai status monyet bangkatan di HSTS termasuklah ancaman terhadap populasi mereka. Kajian ini menemui populasi relik monyet bangkatan yang terpencil melalui tinjauan bot serta sesi temu bual bersama komuniti setempat. Kami mendapati degradasi integriti hutan bakau adalah ancaman utama terhadap spesies monyet ini di HSTS. Kajian ini diharapkan agar memangkin lebih banyak penyelidikan terutamanya penentuan saiz populasi monyet bangkatan kerana ia merupakan maklumat asas dalam penyediaan program pemuliharaan yang efektif.

**Highlights**

- This study discovered a small and isolated relict population of proboscis monkeys through a boat survey approach, which was also derived from interview sessions with local communities.
- We found that the degradation of mangrove forest integrity is a major threat to this population in the Sulaman Lake Forest Reserve (SLFR).
- Immediate and holistic intervention is required to improve the viability of this colobine population in the SLFR amid the ongoing urbanisation particularly the Pan Borneo Highway project.

## INTRODUCTION

Proboscis monkey (*Nasalis larvatus*) is a Bornean endemic primate of the Colobine subfamily (Matsuda *et al*. 2022). This monkey is distributed across three countries within Borneo, namely Malaysia, Indonesia and Brunei (Phillipps & Phillipps 2018). In Sabah, which is a part of Malaysian Borneo, proboscis monkey is locally known as “monyet bangkatan” or “Bayau” (Tangah & Chung 2021). It is treated as a Totally Protected Species in Sabah’s Wildlife Conservation Enactment 1997 (Amendment 2017) (State of Sabah 1997). In 1986, the proboscis monkey was listed as “Vulnerable” before its conservation status changed to “Endangered” in 2000 (IUCN 2021).

The proboscis monkey has captured significant attention from wildlife biologists, who are concerned about its population size (Meijaard & Nijman 2000; Ali *et al*. 2009; Bernard *et al*. 2011; Boonratana 2013; Mawah *et al*. 2015; Matsuda *et al*. 2018). Such information is critical in evaluating the risk of extinction of endangered species in the wild (Wang *et al*. 2016). Proboscis monkey populations in Sabah and Sarawak, are estimated at 5,907 (Sha *et al*. 2008) and 9,586 individuals (Laman & Aziz 2019), respectively. Brunei, on the other hand, is estimated to shelter at least 420 individuals (Salter & MacKenzie 1985). Kalimantan has been estimated to harbour 7,500 individuals (Tisdell & Nantha 2007) but proboscis monkeys in this Indonesian province are scattered due to habitat fragmentation (Rezeki *et al*. 2020).

The overall population of proboscis monkeys is decreasing (IUCN 2021). The decline in the proboscis monkey population in Sabah is linked to habitat fragmentation (Matsuda *et al*. 2018). This worrying population trend has triggered Malaysian authorities to devise long-term conservation efforts in a bid to ensure the survival of proboscis monkeys in Sabah in the future. Sabah has formulated a 10-year action plan (2019–2028) for this primate based on exhaustive discussions between policymakers and biologists (Sabah Wildlife Department 2018). In Sabah, proboscis monkeys have been reported more than in 22 localities, including eight small populations that are at a higher risk of local extinction due to habitat fragmentation (Sha *et al*. 2008).

Large populations (estimated at 4,982 individuals) of proboscis monkeys can be found in the east coast zone (Matsuda *et al*. 2022), where mangrove and riparian forests are extensive. The population of this primate in Klias peninsula (Sabah west coast zone) has been well studied (Bernard *et al*. 2021; Mawah *et al*. 2015; Ali *et al*. 2009). However, literature on this species elsewhere along the western coastal zone is scarce. Multiple records on proboscis monkey occurrences on the west coast of Sabah were based on unverified sources (Sha *et al*. 2008). This signals the urgency to focus on other sites that have not been surveyed (Sabah Wildlife Department 2018), otherwise a small or isolated population might be overlooked, and thus not fully benefit from conservation strategies.

Not all mangrove forests in Sabah are protected because such natural habitat for proboscis monkeys also occurs on state and private land which are meant for future development (Sabah Forestry Department 2020). In a worst-case scenario, local extinction of these populations may go unnoticed by conservationists. Poor management has led to the local extinction of this species in Pulau Kaget Nature Reserve, Indonesia (Meijaard & Nijman 1999).

Sulaman Lake Forest Reserve (SLFR) is in the west coast of Sabah, and is the only Class V Mangrove Forest Reserve in the forestry district of Kota Kinabalu (Sabah Forestry Department 2020). It is segregated, comprising several blocks which partially surround the Sulaman Bay, a major bay in the west of Sabah, and a popular spot among anglers. Governments at both state and federal level are constructing a new highway in this area to create an alternative route from Tuaran to Kota Belud. In December 2019, State Government of Sabah excised a total of 80 ha of forested land from this reserve (Sabah Forestry Department 2020). This indicates that the area along the coastal zone of Sulaman Bay will be developed in the future.

In the 1960s, proboscis monkeys were abundant along the coastal zone of Sulaman Bay (Zaini Uttoh, personal communication, 3 April 2021). Their populations were greatly reduced due to uncontrolled hunting and land-clearing activities. Most hunters were from Muslim communities; the game meats were then sold to non-Muslim customers (Zaini Uttoh, personal communication, 3 April 2021).

Before the gazettement of SLFR in 1984, mangroves were cleared as local settlements expanded, and this was further exacerbated by the operation of a small-scaled charcoal factory in the area. This situation has raised questions about proboscis monkeys in the SLFR. Do they still exist? If so, what is their status in this isolated mangrove ecosystem? To answer these questions, we applied a boat survey method, and complemented it with an interview approach to shed light on the status of proboscis monkeys in the SLFR.

## MATERIALS AND METHODS

### Study Site

**Figure 1:**
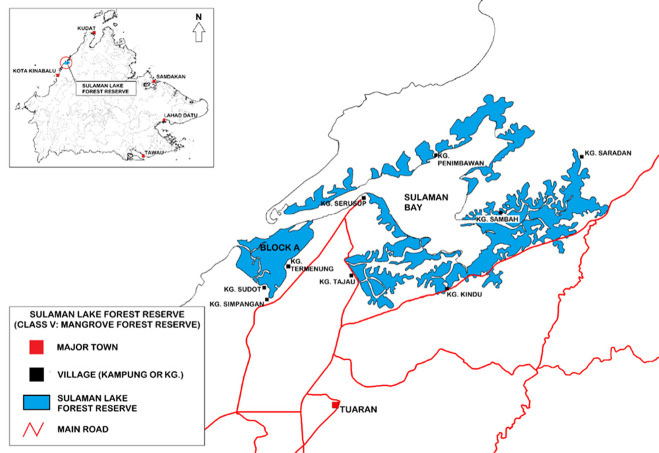
Location of SLFR in Tuaran district. Inset is a map of Sabah, Malaysia (*Source*: Author).

Tuaran is one of the administrative districts in Sabah and adjacent to Kota Kinabalu capital district. The total censused Malaysian population in Tuaran rose by 66%, from 81,215 in 2000 to 134,976 in 2020 (Department of Statistics Malaysia 2020). In 2020, the population density in Tuaran was 120 people per km (Department of Statistics Malaysia 2020). SLFR is in Tuaran district (Fig. 1), with a total gazetted area of 2,555 ha (Sabah Forestry Department 2020).

The function of this SLFR is to supply forest produce, and serve as a recreational or tourism site (Sabah Forestry Department 2020). Mangrove is the dominant forest ecosystem in this reserve, followed by lowland mixed dipterocarp forest and beach forest. The Papat River in the easternmost block (hereafter referred to as Block A) is at least 5 km long during low tide, and approximately 50 m wide at its estuary.

SLFR is adjacent to at least nine local villages (Kampung or Kg., in Malay), namely Kg. Sudot, Kg. Termenung, Kg. Tajau, Kg. Serusup, Kg. Kindu, Kg. Sambah, Kg. Saradan, Kg, Simpangan and Kg. Penimbawan. Some of these villages existed before the gazettement of the reserve. All villages can be accessed via paved road networks. However, most villagers from Kg. Penimbawan prefer boats for their daily transportation, as this is more economical and could reduce travelling time. The local communities of these villages are mostly Muslim of Bajau Sama ethnicity.

### Field Survey on Proboscis Monkey

We applied a boat survey technique to document the occurrences of proboscis monkeys in the SLFR. The survey was conducted between 0800–1800 hours, in 2021 and 2022 (Table 1). The survey team consisted of the boat-man and two observers, namely Mohd Aminur Faiz Suis and Marrynah Matami. We located this species by visual searching with NIKON Monarch 7 binoculars while travelling in a small boat along the coastal line. We excluded other wildlife species from the boat survey. We recorded coordinates with handheld Garmin GPSMAP 64s, and took video or images of the primates with smartphones whenever possible.

In addition to the boat-based survey, we interviewed 58 villagers who frequently fished in the mangroves, or reside adjacent to the forest reserve boundary: Kg. Serusup (14 respondents), Kg. Simpangan (11 respondents), Kg. Kindu (9 respondents), Kg. Penimbawan (7 respondents), Kg. Tajau (7 respondents), Kg. Termenung (4 respondents), Kg. Saradan (3 respondents) and Kg. Sudot (3 respondents). We showed them clear photographs of proboscis monkeys, and a map of the study site. We posed each respondent with the following questions:

1. Are you aware of the presence of proboscis monkeys in the SLFR?
2. Have you encountered this species in the SLFR or in the vicinity of your village?
  2.1 If yes, when was the last time you spotted this species?
  2.2 If yes, where did you encounter this species?
  2.3 If yes, how many individuals have you spotted in a single encounter?

On average, each interview lasted for 10 min. Mohd. Aminur Faiz Suis and Fazidah Ismail, both are local to Kg. Serusup and Kg. Simpangan, respectively, conducted the interviews. These sessions were held between October and December 2023. The interviews were conducted in the Bajau Sama dialect, indicating the authors had established rapport with respondents.

### Assessment on Mangrove Forest Ecosystem

To evaluate the proboscis monkey habitat, we conducted a two-tier forest assessment on mangroves in the SLFR. The first level was assessment using Google Earth Pro version 7.3.6.9796 (64-bit), while the second assessment involved ground truthing. Mangrove forest assessment was conducted in sites where the presence of proboscis monkeys has been verified. We utilised historical satellite imagery on Google Earth Pro to determine the disturbance history of proboscis monkey habitat. We categorised the vegetation into old mangroves and young mangroves at this level. We then conducted ground truthing to validate the first assessment. We measured the diameter at breast height (DBH) of four to five mangrove stems where proboscis monkeys were spotted during the boat survey and identified the species onsite. We also flew a drone (DJI Mavic 2 Pro; Shenzhen DJI Sciences and Technologies Ltd., China) to capture aerial images of the mangrove forest.

There was no vegetation plots established for this study but we utilised unpublished datasets of mangrove vegetation from five ecological research plots that were set up by Joseph Tangah in 2021. These vegetation plots were circular in shape with a 15 m radius each, and represented the disturbed mangrove forest within Block A of the SLFR. Within these plots, all trees with DBH ≥ 10 cm were enumerated and identified.

We also utilised datasets of undisturbed vegetation from five ecological research plots in Sepilok (Mangrove) Forest Reserve (SMFR), Sandakan, Sabah (Tangah *et al*. 2018). Since both vegetation datasets were derived from identical sampling protocols, we made a simple comparison of the DBH distribution classes between disturbed and undisturbed mangrove forests in the SLFR and SMFR, respectively. The latter is a home to a much larger proboscis monkey population on the east coast of Sabah.

## RESULTS

**Figure 2:**
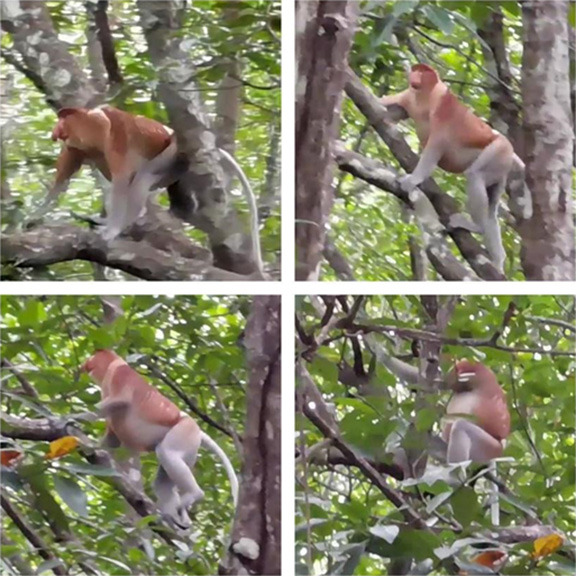
Screenshots from a video of an adult male proboscis monkey captured in SLFR, Sabah, Malaysia.

In total, we found four individuals of proboscis monkeys during the boat survey. We encountered two adult males on the 4 April 2021 in Block A but we were not able to collect photographic evidence. We also spotted a single adult male in Block A on the 23 November 2021 at 1437 hours during the boat survey nearby Kg. Sudot and recorded a short video of this encounter (Fig. 2). The individual was seen climbing mature *Rhizophora apiculata* stems and reaching the leaves. Third encounter happened on the 19 June 2022 and involved a single adult male proboscis monkey.

**Figure 3:**
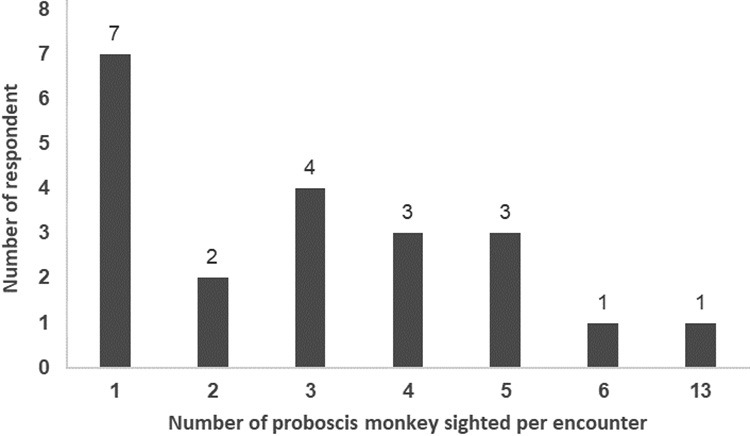
Number of proboscis monkey individuals spotted per encounter by respondents in SLFR, Sabah, Malaysia.

During our survey with residents, only 21 respondents out of 58 had personally encountered proboscis monkeys in the SLFR (Fig. 3). Most of the encounters occurred in Block A, with years of sightings ranging from 2001 to 2023. Interestingly, one respondent who is a former forest ranger sighted a troop of this primate comprising 13 individuals along Papat River in 2016. An educator from Kg. Serusup spotted six individuals in this block in 2019. Sightings of this species outside Block A were reported by respondents from Kg. Kindu, Kg. Saradan and Kg. Penimbawan, but the number of monkeys spotted was fewer than six individuals per encounter between 1990 to 2023. Seven respondents were aware of proboscis monkeys in the vicinity of their villages but had never encountered this primate.

**Figure 4:**
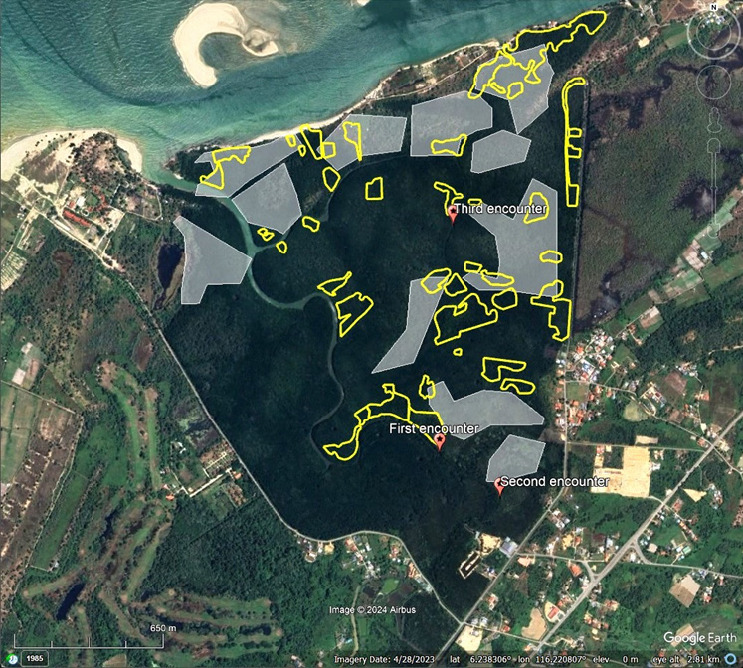
Mosaic of old and young mangroves resulted from episodic land clearance in 1984 (white polygon) and 2003 (yellow polygon) within Block A of SLFR. Location of encounters with proboscis monkeys as indicated on the map (*Source*: Google Earth Pro)

Mangroves in Block A experienced multiple episodes of land clearance activities since 1984, resulting in a mosaic of young and old vegetation (Fig. 4). The block is almost surrounded by paved roads along its perimeter except at the Papat River estuary in the northeast. In 1984, approximately 70 ha of mangrove forest in Block A were cleared. Subsequent clearance occurred in 2003 with 29 ha of mangrove loss. A new local settlement in the northern part of Block A has expanded from just 12 houses in February 2018 to 50 houses in April 2023.

We recorded 25 species of mangroves throughout the fieldwork (Table 2). Young mangroves here were generally small in stature, with DBH and height less than 10 cm and 15 m, respectively. Old-growth mangrove vegetation could mainly be found in the southern part of Block A. Mature mangroves in this reserve grow up to 60 cm in DBH and can attain 35 m in height.

**Figure 5:**
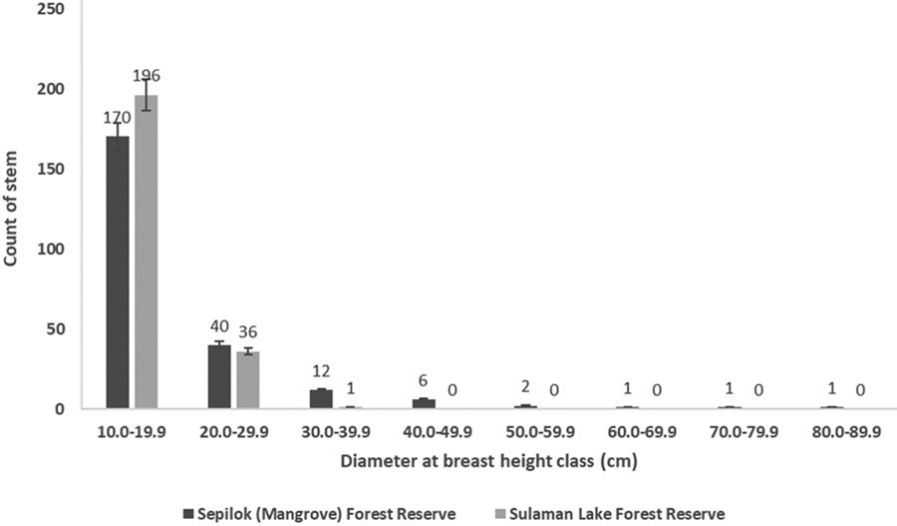
DBH distribution class of enumerated mangroves in SLFR and Sepilok (Mangrove) Forest Reserve, Sabah, Malaysia.

A total of 233 stems were enumerated from five research plots in Block A (Joseph Tangah, unpublished data). The DBH distribution classes of enumerated mangroves indicated a high number of young stems (Fig. 5). In addition, there were no mangrove stems with DBH greater than 40.0 cm within these research plots.

## DISCUSSION

Block A is situated between two proboscis monkey populations in Kota Kinabalu and Kota Belud (Matsuda *et al*. 2022). These populations are separated by extensive road networks and human settlements, with a direct distance of approximately 30 km between the two sites. Based on phylogenetic analysis, current proboscis monkey populations in Sabah are grouped into three major lineages: North, Middle and East clusters (Sabah Wildlife Department 2018). While the genetic structure of the population in the SLFR remains unknown, both the Kota Kinabalu and Kota Belud populations belong to the North cluster.

**Figure 6:**
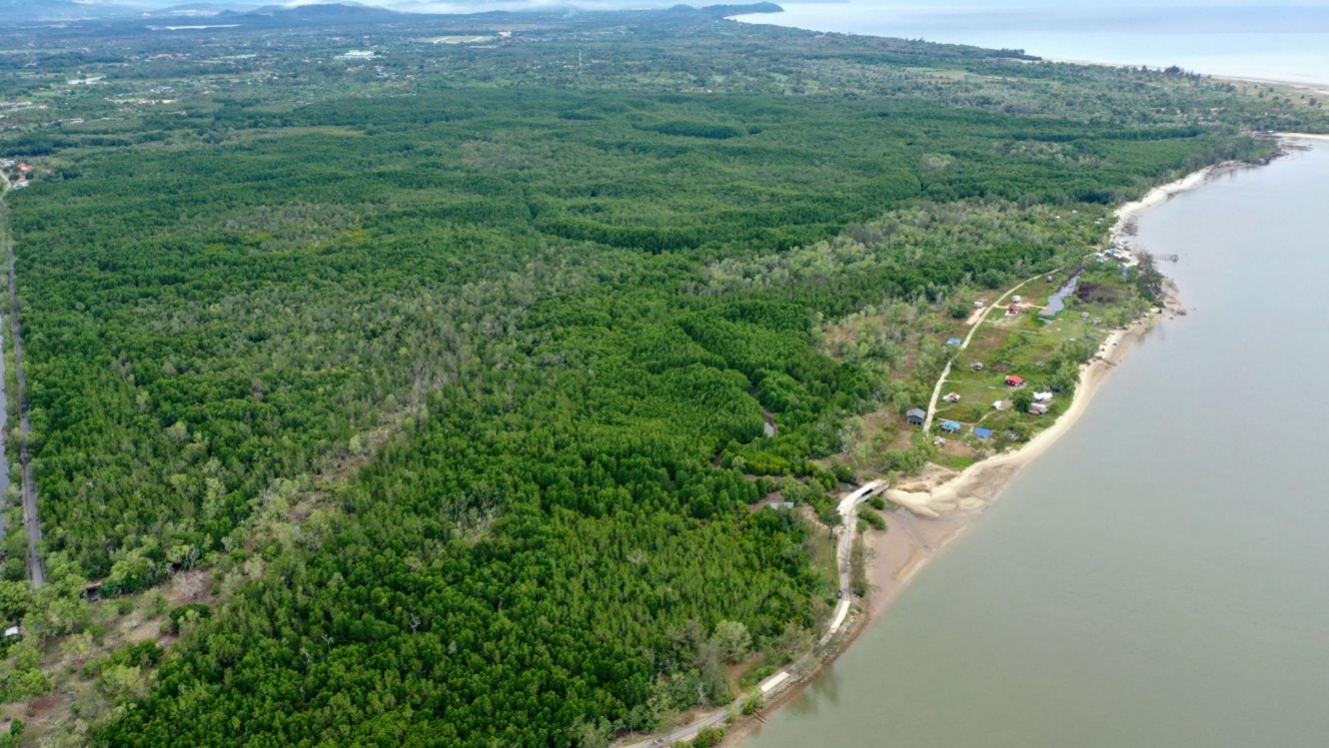
Proboscis monkey’s natural habitat in Block A is surrounded by local villages, paved roads and a golf course. (*Source*: Author)

Block A is physically isolated due to the absence of a contiguous forest canopy with other reserves or mangrove forests (Fig. 6). There have been few reports of proboscis monkeys trying to cross paved roads in Kg. Sudot return to the mangrove forest because of human intervention. Proboscis monkeys are well adapted to cross rivers due to their special body structures (Napier 1985). Individuals from Block A must swim in an open sea for a few kilometres to arrive at another mangrove forest in the west. The long-distance swim is risky because of natural predators (i.e., crocodiles, snakes, monitor lizards) in the area. Moreover, these colobine monkeys prefer to cross rivers at the narrowest width as part of their antipredator behaviour (Yeager 1991). Even if these monkeys survived the westward long-distance swim, other mangrove forests between Tuaran and Kota Kinabalu are not protected as forest reserves. Gazetting these mangrove areas as forest reserves is an uphill battle as it involves privately owned coastal lands. Migrating to other blocks of SLFR in the east might provide a better home ranging area, but anthropogenic pressure along the Sulaman Bay coastal area is gradually increasing.

The extensive riverine forest in lower Kinabatangan floodplain is rich in tree diversity, of which 188 species are consumed by the proboscis monkey (Matsuda *et al*. 2009). Out of 25 mangrove species recorded in Block A, at least 10 species are known to be part of the diet of this monkey (Atmoko 2021; Tangah & Chung 2021). This indicates limited food variety in Block A, which is also low in tree diversity due to the dominant mangrove vegetation. It would be compelling to study the diet of these isolated monkeys in fragmented mangrove habitats.

This study has not determined the minimum population size of proboscis monkeys in the SLFR. However, our respondents sighted up to 13 individuals per encounter in Block A. We are convinced that the population size of this colobine monkey in Block A is less than other small and isolated populations (estimated at 200 individuals) in North Sabah (Matsuda *et al*. 2022). Notably, 63.8% of our respondents had never encountered this species in the SLFR, reflecting its rarity. Frequent rapid survey on proboscis monkeys in other parts of SLFR is highly encouraged because of the reported sightings of this species outside Block A.

Despite limitations faced by proboscis monkeys in Block A, the enumerated mangrove communities are dominated by young stems, which are a prerequisite for active regeneration in mangrove forests (Fan 2008). Active regeneration is also reported in SMFR (Tangah *et al*. 2018), and proboscis monkeys in this reserve are interconnected with populations from both Kinabatangan and Segama through large contiguous wetlands (Matsuda *et al*. 2022). The stark contrast in terms of habitat connectivity has raised our concerns about the future of this species in the SLFR.

Large stems (DBH ≥ 50.0 cm) were found outside the research plots and these mangroves may function as sleeping trees for proboscis monkeys. Colobine monkeys in mangroves of West Kalimantan slept on medium-size stems (mean DBH = 34.3 cm) (Feilen & Marshall 2014). The availability of sleeping trees in the riparian zone of a larger river system is relatively high (Bernard *et al*. 2011; Thiry *et al*. 2016). Conversely, only a few species recorded in Block A have been documented as sleeping trees for this monkey elsewhere in Sabah including *Rhizophora apiculata*, *Bruguiera gymnorhiza*, and *Sonneratia* spp.

Ongoing expansion of local settlements around SLFR is a serious threat to its forest integrity. The urbanisation pace is expected to increase after the completion of Pan Borneo Highway (Work Package 9) which is planned to traverse the SLFR. Mangroves are adversely affected from this rapid development, and a similar issue is also highlighted statewide (Tangah & Chung 2021). Mangrove degradation and destruction is a direct threat to the proboscis monkeys in Block A, as it further reduces the viability of this already fragmented habitat. Furthermore, these colobines are sensitive that any changes in forest vegetation can alter their daily activities (Suwarto *et al*. 2016).

Wildlife poaching is a major conservation issue in many forest reserves and it has affected a wide range of animals in Sabah (Suis *et al*. 2023). Prior to the implementation of Wildlife Conservation Enactment 1997 (State of Sabah 1997), the initial population of proboscis monkeys in the SLFR was severely impacted by human activities. Hunting of proboscis monkeys for meat is often a normal practice among non-Muslim communities in Sabah (Sabah Wildlife Department 2018) and East Kalimantan (Meijaard & Nijman 2000).

Poaching of this primate among predominantly Muslim communities around SLFR is no longer common (Mohd. Aminur Faiz Suis, personal observation). However, we noticed signs of illegal felling of *Lumnitzera littorea* and *Ceriops tagal* in Block A, often used as construction materials in this area. Illegal harvesting of *C. tagal* in the SLFR remains controllable, but young parts of this tree are also consumed by proboscis monkeys (Tangah 2012).

Boat tourism around SLFR is relatively new compared to other sites with abundant proboscis monkeys, such as the lower Kinabatangan floodplain and the Klias peninsula. The presence of fireflies has been documented in the SLFR (Bernard *et al*. 2023) and it is one of the main tourist attractions in this reserve. Even though boat operators knew about proboscis monkeys in Block A, they were often hesitant to include this species in the river cruise itinerary because of the difficulty of visiting the site during low tide, and a sighting is not guaranteed (Mohd. Aminur Faiz Suis, personal observation).

The proboscis monkey is known for avoiding tourism activity (Boonratana 2013), and its abundance is negatively correlated with the number of tourists in the lower Kinabatangan floodplain (Leasor & Macgregor 2014). Tourism development in Sabah is not considered a major threat to this species (Sabah Wildlife Department 2018). However, further research needs to be undertaken to fully elucidate the impact of river-based tourism on a small proboscis monkey population in a fragmented habitat.

## CONCLUSION

The documentation of a small and isolated relict proboscis monkey population in the SLFR is a positive development in an ongoing statewide effort to protect this species. Immediate and holistic intervention is required to improve the viability of this colobine population in the SLFR amid the ongoing urbanisation particularly the Pan Borneo Highway project. We should start with estimating their minimum population size, followed by monitoring at regular intervals.

## Figures and Tables

**Table 1:** Details of boat survey method in SLFR, Sabah, Malaysia.

Date	Time	Coordinates		Distance surveyed (m)	Location

		Start point	End point		
13 February 2021	0800–1200 hours	6°13′15.34″N 116°16′9.02″E	6°12′54.53″N 116°14′43.20″E	6.2 km	Kg. Kindu to Kg. Tajau
14 February 2021	1400–1800 hours	6°13′15.34″N 116°16′9.02″E	6°14′54.57″N 116°17′44.25″E	6.23 km	Kg. Kindu to Kg. Sambah
3 April 2021	1500–1800 hours	6°13′15.34″N 116°16′9.02″E	6°14′53.19″N 116°18′43.51″E	7.69 km	Kg. Kindu to Kg. Saradan
4 April 2021	1500–1800 hours	6°14′1.31″N 116°11′57.08″E	6°13′21.03″N 116°12′37.56″E	2.67 km	Block A
23 November 2021	1300–1630 hours	6°14′1.31″N 116°11′57.08″E	6°13′14.73″N 116°12′46.20″E	2.92 km	Block A
19 June 2022	0800–1530 hours	6°14′1.31″N 116°11′57.08″E	6°13′53.31″N 116°12′39.47″E	1.97 km	Block A

**Table 2:** List of mangrove species recorded in the Block A of SLFR, Sabah, Malaysia.

No.	Family	Species	Local name
1	Rhizophoraceae	*Rhizophora apiculata*	Bangkita or Bakau Minyak
2	Rhizophoraceae	*Rhizophora mucronata*	Bakau Kurap
3	Rhizophoraceae	*Rhizophora stylosa*	Bakau Pasir
4	Rhizophoraceae	*Ceriops tagal*	Tengar
5	Rhizophoraceae	*Ceriops zippeliana*	Tirog
6	Rhizophoraceae	*Bruguiera cylindrica*	Beus
7	Rhizophoraceae	*Bruguiera gymnorhiza*	Putut
8	Rhizophoraceae	*Bruguiera parviflora*	Lenggadai
9	Rhizophoraceae	*Bruguiera hainesii*	Berus Mata Buaya
10	Rhizophoraceae	*Bruguiera sexangula*	Mata Buaya
11	Rhizophoraceae	*Kandelia candel*	Linggayong
12	Acanthaceae	*Avicennia alba*	Api Api Putih
13	Acanthaceae	*Avicennia marina*	Api Api
14	Combretaceae	*Lumnitzera racemosa*	Geriting Putih
15	Combretaceae	*Lumnitzera littorea*	Santing or Geriting Merah
16	Rubiaceae	*Scyphiphora hydrophylacea*	Landing Landing
17	Primulaceae	*Aegiceras corniculatum*	Saka Mata
18	Myrtaceae	*Osbornia octodonta*	Gelam Laut
19	Myrtaceae	*Xylocarpus granatum*	Nyireh
20	Euphorbiaceae	*Excoecaria agallocha*	Buta Buta
21	Lythraceae	*Sonneratia alba*	Perepat
22	Lythraceae	*Sonneratia ovata*	Perepat
23	Pteridaceae	*Acrostichum aureum*	Pakis Piai
24	Acanthaceae	*Acanthus ebracteatus*	Jeruju
25	Arecaceae	*Nypa fruticans*	Nipah
